# Chloro-Furanocembranolides from *Leptogorgia* sp. Improve Pancreatic Beta-Cell Proliferation

**DOI:** 10.3390/md16020049

**Published:** 2018-02-02

**Authors:** Amalia B. Gallardo, Ana R. Díaz-Marrero, José M. de la Rosa, Luis D’Croz, Germán Perdomo, Irene Cózar-Castellano, José Darias, Mercedes Cueto

**Affiliations:** 1 Instituto de Productos Naturales y Agrobiología (IPNA-CSIC), Avenida Astrofísico F. Sánchez, 3, 38206 La Laguna, Tenerife, Spain; amalia.gallardo@umag.cl (A.B.G.); adiazmar@ull.edu.es (A.R.D.-M.); jmrosa@ull.es (J.M.d.l.R.); dariasjose4@gmail.com (J.D.); 2 Departamento de Química, Facultad de Ciencias, Universidad de Magallanes, Avenida Bulnes 01855, Punta Arenas 6200000, Chile; 3 Departamento de Biología Marina y Limnología, Universidad de Panamá, Panama City P.O. Box 3366, Panama; dcrozlc@gmail.com; 4 Smithsonian Tropical Research Institute, STRI, Balboa P.O. Box 0843-03092, Panama; 5 Facultad de Ciencias de la Salud, Universidad de Burgos, 09001 Burgos, Spain; gmperdomo@ubu.es; 6 Instituto de Biología y Genética Molecular, University of Valladolid-CSIC, 47005 Valladolid, Spain; cozarirene@gmail.com

**Keywords:** *Leptogorgia*, cembranolides, furanocembranolides, diketocembranolides, *seco*-furanocembranolides, chloro-furanocembranolides, pancreatic beta-cells

## Abstract

Two new chloro-furanocembranolides (**1**, **2**) and two new 1,4-diketo cembranolides (**3**, **4**) were isolated from the crude extract of *Leptogorgia* sp. together with a new *seco*-furanocembranolide (**5**) and the known *Z*-deoxypukalide (**6**), rubifolide (**7**), scabrolide D (**8**) and epoxylophodione (**9**). Their structures were determined based on spectroscopic evidence. Four compounds: **1**, **2**, **7** and **8** were found to activate the proliferation of pancreatic insulin-producing (beta) cells.

## 1. Introduction

Octocorals of the genus *Leptogorgia* biosynthesize highly oxygenated diterpenoids based on the cembrane skeleton: (a) furanocembranolides into which a γ-lactone subunit is embedded; (b) 1,4-diketocembranoids produced by oxidative cleavage of the furan ring; and (c) *nor*-1,4-diketocembranolides that lack the C-18 isoprenic methyl group [1,2].

**Figure 1:**
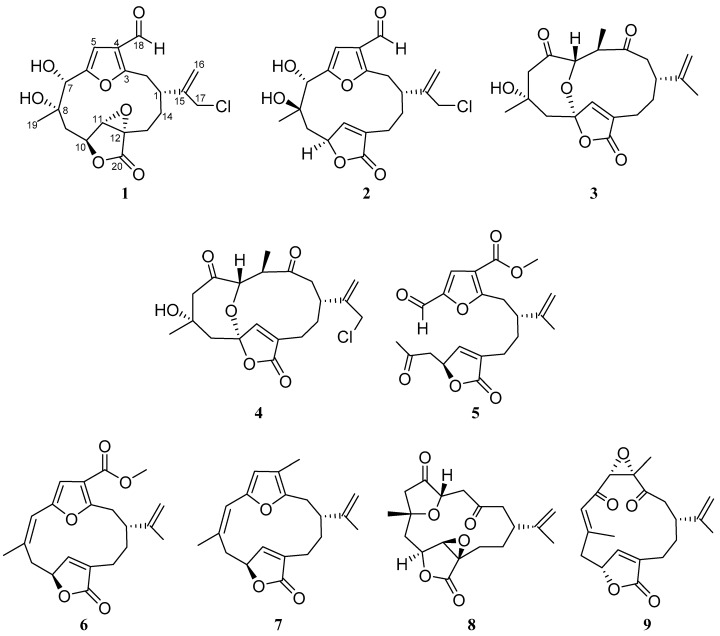
Novel chloro-furanocembranolides (**1**, **2**), 1,4-diketocembranolides (**3**, **4**), *seco*-furanocembranolide (**5**) and the known *Z*-deoxypukalide (**6**), rubifolide (**7**), scabrolide D (**8**) and epoxylophodione (**9**) from *Leptogorgia* sp.

It has been suggested that activation of pancreatic beta-cell proliferation is a strategy to maintain functional beta-cell mass in diabetes mellitus [3]. Regarding this, we have shown that furanocembranoids such as epoxypukalide, pukalide, *Z*-deoxypukalide and leptolide improve beta-cell proliferation [4,5,6]. This prompted us to study a new extract of *Leptogorgia* sp. collected by SCUBA diving off Aleta (Panama), from which compounds **1**–**9** were obtained after flash chromatography followed by HPLC (Figure 1). The unusual halogenated compounds **1** and **2**, together with the known *Z*-desoxypukalide (**6**) [1] and rubifolide (**7**) [7] belong to the furanocembranolide structural class. The remaining six compounds typify three different subclasses that involve several types of rearrangements from a furanocembranolide precursor: (a) **3**, **4** and the previously isolated epoxylophodione (**9**) [8] are 1,4-diketocembranolides, **4** being the only haloderivative of the genus *Leptogorgia* of this subclass; (b) compound **5** is the first *seco*-furanocembranolide isolated from genus *Leptogorgia*, which showed a similar ring scission pattern to *seco*-bipinnatin J, the only *seco*-derivative [9] isolated from *Pseudopterogorgia*; and (c) scabrolide D (**8**) [10] is a *nor*-1,4-diketocembranolide which lacks the C-18 isoprenic methyl group.

## 2. Results

Compound **1** was obtained as an oil whose EIMS spectrum showed peaks at *m*/*z* [M − 1]^+^ 409/411, with relative intensities suggesting one chlorine atom. These peaks correspond to the molecular formula C_20_H_23_ClO_7_ (HREIMS) (*m*/*z* 409.1041 [M − 1]^+^, calcd. for C_20_H_22_^35^ClO_7_ 409.1054). The diterpenic nature of **1** is corroborated by the ^13^C NMR spectrum (in Supplementary Materials), which displayed correlations in the HSQC spectrum indicative of seven quaternary carbons, six methines, six methylenes and one methyl (Table 1). Absorptions for a hydroxyl group at 3476 cm^−1^ and carbonyl groups at 1776 and 1676 cm^−1^ were observed in the IR spectrum. 

Notable ^1^H and ^13^C NMR signals are: an aldehyde group [δ_H-18_ 9.89 (1H, s), δ_C-18_ 184.7], a trisubstituted furane ring [δ_H-5_ 6.70 (1H, s), δ_C-5_ 106.5; δ_C-3_ 161.3; δ_C-6_ 156.0; δ_C-4_ 123.4], a α,β-epoxy-γ-lactone ring [δ_H-11_ 3.73 (1H, s), δ_C-11_ 62.9; δ_C-20_ 172.1; δ_H-10_ 4.85 (1H, dd, *J* = 5.2, 11.3 Hz), δ_C-10_ 74.3 and δ_C-12_ 60.6] and a chloromethylene [δ_H-17_ 4.18 (2H, br s) y δ_C-17_ 46.6].

Connectivity information obtained from COSY, HSQC and HMBC experiments unambiguously determined the planar structure of compound **1** as a furanocembranolide, containing a C-18 oxidized to aldehyde, a vicinal diol at C-7–C-8, a C-10–C-20 α,β-epoxy-γ-lactone moiety and a chloroisopropenyl group at C-1.

**Figure 2:**
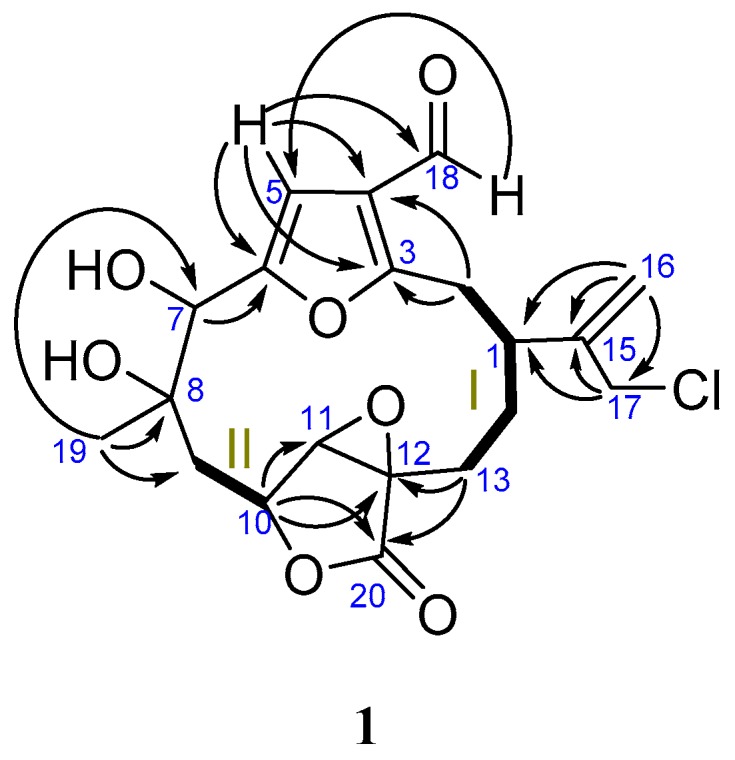
^1^H-^1^H-COSY (**—**), HMBC (→) correlations of **1**.

^1^H-^1^H-COSY experiments established two spin systems: H_2_-2–H_2_-13 (fragment **I**) and H_2_-9–H-10 (fragment **II**) (Figure 2). The HMBC correlations H_2_-16/C-17, C-15, C-1 and H_2_-17/C-16, C-15, C-1 locate a chloroisopropenyl group at C-1 of fragment **I**, whereas the correlations H_3_-19/C-7, C-8 and C-9 allowed us lengthen fragment **II** by adding a vicinal dihydroxyl moiety bonded to C-9. The north end of both fragments **II** and **I** are connected together by insertion of a furane ring, in agreement with the HMBC correlations H-5/C-3, C-4, C-6 and H_2_-2 with C-4. An epoxy lactone binds the southern ends of fragments **I** and **II** between C-13 and C-10, as deduced from the HMBC correlations H-10/C-11, C-12, C-20; H_2_-13/C-20, C-11, C-12 and H_2_-14 with C-12. Thus, the structure of **1**, with nine degrees of unsaturation, has been established.

Compound **2** was obtained as an oil whose EIMS spectrum showed a molecular ion at *m*/*z* [M]^+^ 394/396, with an isotopic pattern for a chlorine atom in the molecular formula C_20_H_23_ClO_6_ (HREIMS) (*m*/*z* 394.1181 [M]^+^, calcd. for C_20_H_23_^35^ClO_6_ 394.1183). The ^13^C NMR spectrum and correlations in the HSQC spectrum indicated seven quaternary carbons, six methines, six methylenes and one methyl (Table 1). Absorptions for a hydroxyl group at 3558 cm^−1^ and carbonyl group at 1747 cm^−1^ were observed in its IR spectrum.

^1^H and ^13^C NMR data resemble those of **1**. The principal differences lie in the chemical shifts of H-7, H_2_-9, C-9 and C-19, which were: δ_H-7_ 4.59 (1H, s), δ_H-9_ 1.89 (1H, m); 2.59 (1H, dd); δ_C-9_ 43.0 and δ_C-19_ 19.6, compared with those of **1** (Table 1). These shift values suggested that compounds **1** and **2** differ in the configuration of C-7 and C-8. Also, their molecular formulas diverge by 16 amu of oxygen. This suggests that the γ-lactone ring system is devoid of the epoxide ring on **2**. This absence is confirmed by the new signals observed in the ^1^H and ^13^C NMR spectra for a proton (δ_H-11_ 5.86 (1H, s)) of a disubstituted olefin (δ_C-11_ 148.6; δ_C-12_ 136.2). Thus, the structure of **2** with nine degrees of unsaturation was established as shown in Figure 1 by COSY, HSQC and HMBC experiments.

**Figure 3:**
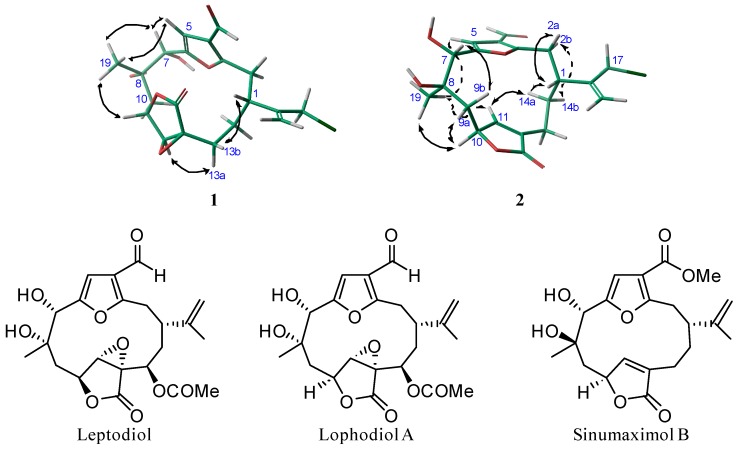
Selected NOE effects (↔) of **1** and **2** and leptodiol, lophodiol A and sinumaximol B.

The relative configurations of compounds **1** and **2** were ascertained by NOESY experiments, molecular mechanics [11], chemical shift studies and comparison of their spectroscopic data with those of previously described cembranoids, leptodiol [1], lophodiol A [12] and sinumaximol B [13] (Figure 3).

In compound **1**, the observed NOEs of H-5 with H-7 and H_3_-19, together with those of H-7 with H_3_-19 suggested that the adjacent hydroxyl groups at C-7–C-8 should be in a *cis* relationship. In compound **2**, the observed NOEs of H_3_-19 with H-5 and H-10 indicate that these protons and Me-19 must be on same side of the molecule, whereas the NOEs of H_3_-19 with H-9a and of H-7 with H-9b indicates that H-7 and Me-19, so the vicinal diols on C-7–C-8, have a *trans-*relationship. Therefore, the relative configuration of C-8 is opposite to that on compound **1**.

The configurations of C-7–C-8 vicinal diols were corroborated by comparison of the ^1^H and ^13^C NMR chemical shifts around the diol moiety C-7–C-8 of compounds **1** and **2** with those of the related diols leptodiol, lophodiol A and sinumaximol B, shown in Table 2. The chemical shifts of C-19 and H-7 of compounds **1** and **2** present strong differences (Δδ_C-19_ = 2.8 ppm and Δδ_H-7_ = 0.7). The chemical shifts of C-19 and H-7 of leptodiol (δ_C-19_ 22.7; δ_H-7_ 5.12) and lophodiol A (δ_C-19_ 22.7; δ_H-7_ 5.24), both with the C-7–C-8 diols in an α-*cis*-relationship, are very similar to those of compound **1**. Whereas the chemical shifts of C-19 and H-7 of sinumaximol B (δ_C-19_ 19.8; δ_H-7_ 4.52), whose C-7–C-8 diols show a *trans-*relationship, present strong differences (upfield Δδ_C-19_ ≈ 2.6 ppm and downfield Δδ_H-7_ ≈ 0.8) which are very similar to those presented by compound **2** in comparison to compound **1**.

In compound **1**, a striking ^1^H NMR signal is the singlet observed for H-11 (δ 3.73, s) due to the roughly 90° dihedral angle formed between H-10 and H-11. This results in a small *J*_H-10, H-11_ that confirms the relative configuration of C-10 and C-11 as represented in the energetically favourable conformation shown in Figure 3.

Finally, in compound **1**, the relative configuration of C-1 was secured by the NOESY correlation of H-13b with H-1 and H-13a with H-11, as shown in the 3D model in Figure 3. In compound **2**, the observed NOEs of H-1 with H-2a and H-14a, as well as of H-14a with H-11 and of H-2b with H-14b, indicate that the isopropenyl group of **2** is situated on the alpha side of the molecule. Therefore, both compounds belong to the furanocembranolide of the α-series and their relative configurations are: 1R*, 7S*, 8S*, 10S*, 11S* and 12S* for compound **1** and 1R*, 7S*, 8R* and 10S* for compound **2**.

Compound **3** was obtained as an oil whose EIMS spectrum showed a peak at *m*/*z* 362 [M]^+^, which corresponds to the molecular formula C_20_H_26_O_6_ (HREIMS) (*m*/*z* 362.1737 [M]^+^, calcd. for C_20_H_26_O_6_ 362.1729). These data are in agreement with the ^13^C NMR spectrum, which displayed correlations in the HSQC spectrum indicative of seven quaternary carbons, four methines, six methylenes and three methyls. Absorptions for a hydroxyl group at 3475 cm^−1^ and carbonyl groups at 1751, 1721 and 1701 cm^−1^ were observed in their IR spectrum. According to the degree of unsaturation given by the ^13^C NMR data, **3** must be a tricyclic compound.

In the ^1^H and ^13^C NMR experiments (Table 3), signals were found for: α,β-unsaturated-γ-lactone ring [δ_H-11_ 6.76 (1H, s), δ_C-11_ 155.9; δ_C-20_ 170.6; δ_C-10_ 90.5]; isopropenyl group [δ_H-16_ 4.68 (1H, br s), δ_H-16_ 4.70 (1H, br s); δ_C-16_ 109.7] and [δ_H-17_ 1.69 (3H, s); δ_C-17_ 20.9]; methyl group on a quaternary carbon bonded to oxygen [δ_H-19_ 1.37 (CH_3_, s) and δ_C-8_ 74.3]; oximethine proton bonded to a secondary carbon [δ_H-5_ 3.31 (1H, d, 10.4 Hz) and δ_C-5_ 60.8] and two carbonyls [δ_C-3_ 215.4 and δ_C-6_ 203.5].

**Figure 4:**
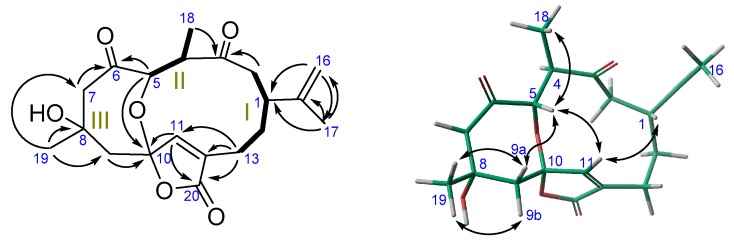
^1^H-^1^H-COSY (**—**), HMBC (→) and selected NOE effects (↔) of **3**.

^1^H-^1^H-COSY experiments established two spin systems: H_2_-2–H_2_-13 (fragment **I**) and H_3_-18–H-5 (fragment **II**) (Figure 4). The HMBC correlations of H_2_-16/C-1, C-17 and H_3_-17/C-16, C-15, C-1 locates the isopropenyl group at C-1 in fragment **I**, whereas the HMBC correlations of H_2_-2 and H_3_-18 with C-3 connects fragments **I** and **II** through C-3. The HMBC correlations H_3_-19/ C-7, C-8, C-9, along with those of H-5 and H_2_-7 with C-6, allowed us to extend fragment **II** by connecting it with substructure **III** through C-6. Both ends of the fragment **I** and substructure **III** (C-6–C-9) are connected by inserting an α,β-unsaturated γ-lactone bonded to C-13 and C-9, respectively, due to the HMBC correlations H_2_-13/C-20, C-11, C-12 and those of H-11/C-10 and H_2_-9/C-10, C-11. The quaternary feature of C-10 comes from the oxygen linkage between C-5 and C-10 supported by an HMBC correlation of H-5 with C-10. Therefore, the tricyclic structure of **3** has been established as depicted in Figure 4.

Compound **4** was obtained as an oil whose EIMS spectrum showed peaks at *m*/*z* 396/398 [M]^+^, which correspond to the formula C_20_H_25_ClO_6_ (HREIMS) (*m*/*z* 396.1324 [M]^+^, calcd. for C_20_H_25_O_6_^35^Cl, 396.1340). Considering the HSQC correlations, signals observed in the ^13^C NMR spectrum indicate seven quaternary carbons, four methines, seven methylenes and two methyls. Absorptions for a hydroxyl group at 3420 cm^−1^ and carbonyl groups at 1727, 1690 and 1647 cm^−1^ were observed in their IR spectrum. 

^1^H and ^13^C NMR data (Table 3) resemble those of **3**. The molecular formula of **4** showed that one proton of **3** is substituted by a chlorine atom in **4**. Its corresponding data from both ^1^H and ^13^C NMR reveal that the substitution is situated on the isopropenyl appendage, where the methyl group of **3** changed to chloromethylene in **4**. This substitution was confirmed by the fragment (*m*/*z* 321.1335 [M − C_3_H_4_Cl]^+^, calcd. for C_17_H_21_O_6_, 321.1338) observed in HREIMS. The planar structure of **4** was confirmed as the 17-chloro derivative of compound **3** by COSY, HSQC and HMBC experiments.

NOESY experiments, studies of coupling constants and molecular mechanics calculations suggest that **3** and **4** have the same relative stereochemistry (Figure 1). In both compounds, NOEs were observed for H-5 with H-9a and H-11 and for H_3_-19 with H-9a and H-9b, defining a relative configuration for C-5 and C-10 and establishing H_3_-19 on C-8 in a *pseudo*-equatorial disposition and therefore coplanar to H-5. Also, the large coupling constants of H-5 (*J* = 10.4 Hz) in **3** and H-5 (*J* = 10.3 Hz) in **4** indicate that H-5 and H-4 are *trans*-diaxial, as the observed NOE between H-5 and H_3_-18 corroborates. Finally, the NOE observed between H-11 and H-1 places the isopropenyl group on the opposite side to the Me-18. Therefore, the overall relative configuration for **3** and **4** should be 1R*, 4R*, 5S*, 8R* and 10S*.

Compound **5** was obtained as an oil with an EIMS spectrum peak at *m*/*z* 388 [M]^+^, which corresponds to the molecular formula C_21_H_24_O_7_ (HREIMS) (*m*/*z* 388.1524 [M]^+^, calcd. for C_21_H_24_O_7_, 388.1522). These data are in agreement with the ^13^C NMR spectrum, which displayed correlations in the HSQC spectrum indicating eight quaternary carbons, five methines, five methylenes and three methyls (Table 3). Absorptions for carbonyl groups at 1655, 1650 and 1638 cm^−1^ were noted in the IR spectrum. 

In addition to the ^1^H and ^13^C NMR data registered for an isopropenyl group, a furan ring and a α,β-unsaturated-γ-lactone, other notable key signals were detected for the following functional groups: aldehyde [δ_H-7_ 9.54 (1H, s), δ_C-7_ 177.1]; methyl ketone [δ_H-19_ 2.21 (3H, s), δ_C-8_ 204.3] and methyl ester [δ_H-21_ 3.86 (3H, s), δ_C-18_ 162.9]. The 21 carbon atoms given by the molecular formula suggested that the isoprenic methyl group C-18 of a regular furanocembranolide is oxidized to a methyl ester and that the methyl-ketone and the aldehyde might come from the oxidative cleavage of the C-7–C-8 bond. According to the connectivity information from experiments COSY, HSQC and HMBC, compound **5** should be a *seco*-furanocembranolide.

**Figure 5:**
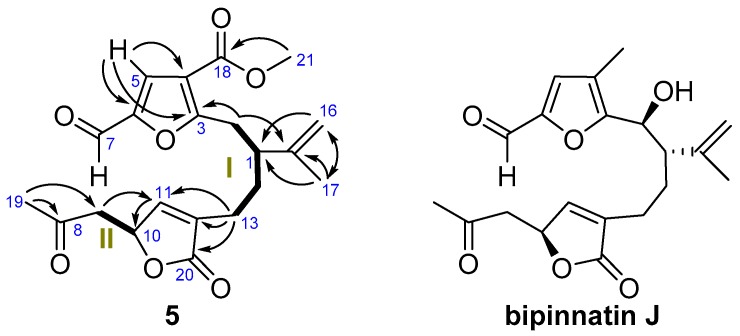
^1^H-^1^H-COSY (—) and HMBC (→) correlations of **5** and bipinnatin J.

^1^H-^1^H-COSY experiments established two spin systems: H_2_-2–H_2_-13 (fragment **I**) and H_2_-9–H-10 (fragment **II**) (Figure 5). The HMBC correlations of H_2_-16/C-1, C-17 and H_3_-17/C-16, C-15, C-1 locate the isopropenyl group at C-1 of fragment **I** and also those of H-5/C-3, C-4, C-6 and H_2_-2 with C-3 connected fragment **I** at the furan ring. The HMBC correlations of H_2_-13/C-11, C-12, C-20 and H-11/C-10 and H_2_-9/C-11 showed that fragments **I** and **II** are linked through an α,β-unsaturated-γ-lactone. HMBC correlations of H_3_-19/C-8, C-9 allowed us to place the methyl-ketone. Finally, the aldehyde must be located at C-6, in good agreement with the chemical shift observed for the aldehyde group of *seco*-bipinnatin J (δ_H-7_ 9.52 (1H, s), δ_C-7_ 177.5), the only *seco*-derivative [9] isolated from *Pseudopterogorgia*, which showed the same scission pattern.

Compound **5** is the first *seco*-furanocembranolide isolated from genus *Leptogorgia*. *Z*-deoxypukalide [1], also isolated in this work, can be considered a biogenetic precursor of **5** by oxidative cleavage of the corresponding Δ^7,8^. Since *Z*-deoxypukalide belongs to the α-cembranolide series, we assign the same relative configurations 1R*, 10S* to **5**.

It should be expected that compounds **1**–**5** belong to the same enantiomeric series as *Z*-deoxypukalide, (**6**) whose absolute configuration we have previously determined using an NMR-based method using Pirkle’s reagent [1].

### Activation of Pancreatic Beta-Cell Proliferation

Several strategies have been proposed to recover functional beta-cell mass loss in diabetes mellitus onset; one of them is to activate beta-cell proliferation [3]. In previous work, we showed that furanocembranolides such as epoxypukalide, pukalide, *Z*-deoxypukalide and leptolide augment beta-cell proliferation [4,5,6]. In order to acquire detailed knowledge of the proliferation effect induced by furanocembranolides, compounds **1**, **2** and rubifolide (**7**) were used to treat synchronized INS-1 cells and proliferation was then measured. INS-1 cells were preincubated with 0.1 μM of each product and proliferation was measured by BrdU incorporation (Table 4), showing a 2–3-fold increase in proliferation. Although it is difficult to reach a conclusion regarding the functional groups that could modulate this proliferation activity, these results also support chloro-furanocembranolides being potential activators of pancreatic beta-cell proliferation.

Furthermore, synchronized INS-1 cells were treated with the *nor*-cembranolide, scabrolide D (**8**), (Table 4), showing a 2.8 ± 0.69-fold change above untreated cells (1.0). We therefore consider it of interest in searching for compounds of the furanocembranolide and *nor*-1,4-diketocembranolide families, in order to develop a new class of antidiabetic agents.

## 3. Experimental Section

### 3.1. General Experimental Procedures

Optical rotations were measured on a Perkin-Elmer model 343 Plus polarimeter (Perkin-Elmer, Rodgau, Germany) using a Na lamp at 20 °C. IR spectra were recorded on a Perkin-Elmer 1650/FTIR spectrometer (Perkin-Elmer, Rodgau, Germany). ^1^H NMR and ^13^C NMR, HSQC, HMBC and COSY spectra were measured employing a Bruker AMX 500 instrument (Bruker, Karlsruhe, Germany) operating at 500 MHz for ^1^H NMR and at 125 MHz for ^13^C NMR. All ^13^C and ^1^H NMR spectra were internally referenced to the residual solvent signal (CDCl_3_: δ_C_ 77.0 ppm, δ_H_ 7.25 ppm). Two-dimensional NMR spectra were obtained using the standard Bruker software (TOpSpin 2.1, Bruker, Karlsruhe, Germany). The EIMS data were obtained on a Waters Vg-Micromass spectrometer (Waters, Manchester, UK) model Zab 2F. HPLC separations were performed on an Agilent 1200 Series Quaternary LC system (Agilent Technologies, Waldbronn, Germany) apparatus equipped with a UV detector (DAD G1315D, Agilent Technologies, Waldbronn, Germany) and an Ascentis^®^ C18 semi-preparative column (5 μm, 25 cm × 21.2 mm, Supelco, Bellefonte, PA, USA) eluted with CH_3_CN-H_2_O mixtures. Size-exclusion chromatography used Sephadex LH-20 as stationary phase and hexane-MeOH-CH_2_Cl_2_ (3:1:1) as solvent system. The spray reagent used to develop TLC plates was H_2_SO_4_-H_2_O-AcOH (1:4:20).

### 3.2. Collection, Extraction and Isolation

*Leptogorgia* sp. was collected by SCUBA diving off Aleta (Panama) at −10 m. A voucher specimen has been deposited at the Smithsonian Tropical Research Institute (Panama City, Panama) with code 200708. Specimens of *Leptogorgia* sp. (458.3 g) were extracted with acetone at room temperature and were concentrated to give a dark gum (10.1 g). C-18 reversed-phase flash chromatography of the crude extract gave fractions 4 (290.4 mg; 2:3 H_2_O/MeOH) and 5 (1119.1 mg; 1:4 H_2_O/MeOH) containing cembranolides, as indicated by their ^1^H NMR spectra. Fraction 4 was further chromatographed by molecular exclusion LH-20 to give two sub-fractions of interest, 4_1_ (39.4 mg) and 4_5_ (18.6 mg). C-18 reversed-phase HPLC of 4_1_ using a gradient from H_2_O-CH_3_CN (7:3) to CH_3_CN (100%) afforded compound **1** (1.3 mg, t_R_ 61 min) and scabrolide D (**8**) (6.5 mg, t_R_ 64 min). From sub-fraction 4_5_, compounds **2** (5.4 mg; t_R_ 101.5 min) and **4** (0.5 mg; t_R_ 83 min) were separated after C-18 reversed-phase HPLC using a gradient from H_2_O-CH_3_CN (7:3) to CH_3_CN (100%). Fraction 5 was chromatographed by molecular exclusion LH-20 to give five sub-fractions of interest: 5_1_ (87.6 m), 5_2_ (89.5 mg), 5_3_ (82.9 mg), 5_4_ (59.1 mg) and 5_5_ (50.3 mg). All these fractions were chromatographed separately by C-18 reversed-phase HPLC using a gradient from H_2_O-CH_3_CN (7:3) to CH_3_CN (100%), to afford compounds **3** (1.6 mg; t_R_ 83.1 min), **5** (2.1 mg; t_R_ 52.3 min), *Z*-deoxypukalide (**6**) (5.7 mg; t_R_ 111.2 min), rubifolide (**7**) (6.0 mg; t_R_ 74 min) and epoxylophodione (**9**) (0.4 mg; t_R_ 82.3 min).

Compound **1**: Colourless oil; ${\left[\alpha \right]}_{D}^{20}$ −20.7 (*c* 0.03, CH_2_Cl_2_); IR (film) ν_max_ 3476, 2932, 1776, 1676 cm^−1^; ^1^H (500 MHz, CDCl_3_) δ 1.35 (3H, s, H-19), 1.42 (1H, m, H-14a), 1.45 (1H, m, H-13a), 1.51 (1H, m, H-9), 1.87 (1H, dd, *J* = 5.2, 15.0 Hz, H-9), 2.04 (1H, m, H-14b), 2.44 (1H, dd, *J* = 11.5, 14.7 Hz, H-13b), 3.15 (2H, m, H-2), 3.39 (1H, m, H-1), 3.73 (1H, s, H-11), 4.18 (2H, br s, H-17), 4.85 (1H, dd, *J* = 5.2, 11.3 Hz, H-10), 5.29 (1H, s, H-7), 5.37 (1H, s, H-16a), 5.47 (1H, s, H-16b), 6.70 (1H, s, H-5), 9.89 (1H, s, H-18); ^13^C NMR (125 MHz CDCl_3_) δ 22.4 (CH_3_, C-19), 22.5 (CH_2_, C-13), 30.4 (CH_2_, C-14), 32.9 (CH_2_, C-2), 37.6 (CH, C-1), 40.5 (CH_2_, C-9), 46.6 (CH_2_, C-17), 60.6 (C, C-12), 62.9 (CH, C-11), 74.0 (CH, C-7), 74.3 (C, C-8), 74.3 (CH, C-10), 106.5 (CH, C-5), 118.0 (CH_2_, C-16), 123.4 (C, C-4), 144.2 (C, C-15), 156.0 (C, C-6), 161.3 (C, C-3), 172.0 (C, C-20), 184.7 (CH, C-18); EIMS *m*/*z* 409/411 [M − 1]^+^, 395/397 [M − CH_3_]^+^, 393/395 [M − OH]^+^, 355 [M − C_3_H_4_Cl]^+^; HREIMS *m*/*z* [M − 1]^+^ 409.1041 (calcd. for C_20_H_22_^35^ClO_7_ 409.1054), 411.1027 (calcd. for C_20_H_22_^37^ClO_7_, 411.1025), 395.0887 (calcd. for C_19_H_20_^35^ClO_7_, 395.0898), 395.1069 (calcd. for C_20_H_22_^37^ClO_6_, 395.1075).

Compound **2**: Colourless oil; ${\left[\alpha \right]}_{D}^{20}$ −11.0 (*c* 0.10, CH_2_Cl_2_); IR (film) ν_max_ 3558, 2935, 1747, 1674 cm^−1^; ^1^H (500 MHz, CDCl_3_) δ 1.41 (3H, s, H-19), 1.60 (1H, ddd, *J* = 2.8, 2.8, 15.1 Hz, H-14a), 1.89 (1H, dd, *J* = 11.7, 14.8 Hz, H-9b), 2.02 (1H, m, H-14b), 2.14 (1H, m, H-13a), 2.35 (1H, ddd, *J* = 2.8, 11.9, 15.1 Hz, H-13b), 2.46 (1H, dddd, *J* = 2.2, 2.5, 9.5, 11.7 Hz, H-1), 2.59 (1H, dd, *J* = 4.1, 14.8 Hz, H-9a), 2.97 (1H, dd, *J* = 2.5, 14.8 Hz, H-2a), 3.19 (1H, dd, *J* = 11.9, 15.1 Hz, H-2b), 4.12 (2H, br s, H-17), 4.59 (1H, s, H-7), 4.97 (1H, m, H-10), 5.19 (1H, s, H-16), 5.36 (1H, s, H-16), 5.86 (1H, s, H-11), 6.80 (1H, s, H-5), 9.97 (1H, s, H-18); ^13^C NMR (125 MHz, CDCl_3_) δ 19.6 (CH_3_, C-19), 21.7 (CH_2_, C-13), 30.2 (CH_2_, C-14), 32.7 (CH_2_, C-2), 39.7 (CH, C-1), 43.0 (CH_2_, C-9), 47.2 (CH_2_, C-17), 73.6 (C, C-8), 75.6 (CH, C-7), 78.4 (CH, C-10), 106.5 (CH, C-5), 117.0 (CH_2_, C-16), 122.7 (C, C-4), 136.2 (C, C-12), 146.9 (C, C-15), 148.6 (CH, C-11), 154.3 (C, C-6), 162.1 (C, C-3), 173.5 (C, C-20), 184.3 (CH, C-18); EIMS *m*/*z* 394/396 [M]^+^, 377/379 [M − OH]^+^; HREIMS *m*/*z* [M]^+^ 394.1181 (calcd. for C_20_H_23_O_6_^35^Cl, 394.1183), 396.1162 (calcd. for C_20_H_23_O_6_^37^Cl, 396.1154), 377.1146 (calcd. for C_20_H_22_O_5_^35^Cl, 377.1156), 379.1133 (calcd. for C_20_H_22_O_5_^37^Cl, 379.1126).

Compound **3**: Colourless oil; ${\left[\alpha \right]}_{D}^{20}$ +10.0 (*c* 0.16, CH_2_Cl_2_); IR (film) ν_max_ 3475, 2933, 1751, 1721, 1701 cm^−1^; ^1^H (500 MHz, CDCl_3_) δ 1.04 (3H, d, *J* = 6.6 Hz, H-18), 1.37 (3H, s, H-19), 1.69 (3H, s, C-17), 1.89 (2H, m, H-14), 1.92 (1H, m, H-9b), 2.27 (2H, ddd, *J* = 5.4, 9.3, 14.3 Hz, H-13a), 2.34 (1H, d, *J* = 15.1 Hz, H-9a), 2.44 (1H, m, H-2a), 2.46 (1H, m, H-2b), 2.50 (1H, m, H-13b), 2.64 (1H, ddd, *J* = 4.2, 7.1, 7.1 Hz, H-1), 2.68 (2H, m, H-7), 2.89 (1H, ddd, *J* = 6.6, 6.6, 10.6 Hz, H-4), 3.31 (1H, d, *J* = 10.4 Hz, H-5), 4.68 (1H, br s, H-16a), 4.70 (1H, br s, H-16b), 6.76 (1H, s, H-11); ^13^C NMR (125 MHz, CDCl_3_) δ 17.2 (CH_3_, C-18), 20.9 (CH_3_, C-17), 24.0 (CH_2_, C-13), 30.1 (CH_3_, C-19), 31.7 (CH_2_, C-14), 39.7 (CH, C-1), 41.9 (CH, C-4), 45.6 (CH_2_, C-9), 49.7 (CH_2_, C-2), 55.9 (CH_2_, C-7), 60.8 (CH, C-5), 74.3 (C, C-8), 90.5 (C, C-10), 109.7 (CH_2_, C-16), 131.4 (C, C-12), 149.4 (C, C-15), 155.9 (CH, C-11), 170.5 (C, C-20), 203.5 (C, C-6), 215.4 (C, C-3); EIMS *m*/*z* 362 [M]^+^, 347 [M − CH_3_]^+^, 345 [M − OH]^+^; HREIMS *m*/*z* [M]^+^ 362.1737 (calcd. for C_20_H_26_O_6_, 362.1729), [M − CH_3_]^+^ 347.1509 (calcd. for C_19_H_23_O_6_, 347.1495), 345.1708 (calcd. for C_20_H_25_O_5_, 345.1702).

Compound **4**: Colourless oil; ${\left[\alpha \right]}_{D}^{20}$ +66.0 (*c* 0.05, CH_2_Cl_2_); IR (film) ν_max_ 3420, 2956, 1727, 1690, 1647 cm^−1^; ^1^H (500 MHz, CDCl_3_) δ 1.04 (3H, d, *J* = 6.6 Hz, H-18), 1.37 (3H, s, H-19), 1.90 (1H, dd, *J* = 2.6, 15.4 Hz H-9b), 2.00 (2H, m, H-14), 2.28 (1H, m, H-13a), 2.32 (1H, d, *J* = 15.1 Hz, H-9b), 2.49 (1H, m, H-13b), 2.53 (2H, m, H-2), 2.69 (2H, m, H-7), 2.90 (1H, m, H-1), 2.90 (1H, m, H-4), 3.31 (1H, d, *J* = 10.3 Hz, H-5), 4.03 (2H, m, H-17), 5.00 (1H, s, H-16b), 5.20 (1H, dd, *J* = 0.9, 0.9 Hz, H-16a), 6.79 (1H, s, H-11); ^13^C NMR (125 MHz, CDCl_3_) δ 17.3 (CH_3_, C-18), 22.8 (CH_2_, C-13), 30.1 (CH_3_, C-19), 31.5 (CH_2_, C-14), 35.4 (CH, C-1), 41.7 (CH, C-4), 45.6 (CH_2_, C-9), 47.6 (CH_2_, C-17), 49.9 (CH_2_, C-2), 55.9 (CH_2_, C-7), 60.9 (CH, C-5), 74.3 (C, C-8), 90.3 (C, C-10), 114.7 (CH_2_, C-16), 131.1 (C, C-12), 149.3 (C, C-15), 156.0 (CH, C-11), 171.6 (C, C-20), 205.8 (C, C-6), 215.3 (C, C-3); EIMS *m*/*z* 396/398 [M]^+^, 378/380 [M − H_2_O]^+^, 361 [M − Cl]^+^, 321 [M − C_3_H_4_Cl]^+^; HREIMS *m*/*z* [M]^+^ 396.1324 (calcd. for C_20_H_25_O_6_^35^Cl, 396.1340), 398.1315 (calcd. for C_20_H_25_O_6_^37^Cl, 398.1310), 361.1642 (calcd. for C_20_H_25_O_6_, 361.1651), 321.1335 (calcd. for C_17_H_21_O_6_, 321.1338).

Compound **5**: Colourless oil; ${\left[\alpha \right]}_{D}^{20}$ −2.11 (*c* 0.21, CH_2_Cl_2_); IR (film) ν_max_ 2100, 1655, 1650, 1638 cm^−1^; ^1^H (500 MHz, CDCl_3_) δ 1.64 (1H, m, H-14), 1.68 (3H, s, H-17), 1.72 (1H, m, H-14), 2.16 (1H, m, H-13a), 2.21 (3H, s, H-19), 2.25 (1H, m, H-13b), 2.63 (1H, dd, *J* = 7.3, 17.7 Hz, H-9a), 2.70 (1H, m, H-1), 2.99 (1H, dd, *J* = 6.7, 17.7 Hz, H-9b), 3.12 (1H, dd, *J* = 6.6, 14.2 Hz, H-2), 3.22 (1H, dd, *J* = 8.5, 14.2 Hz, H-2), 3.86 (3H, s, H-21), 4.64 (1H, br s, H-16a), 4.73 (1H, dd, *J* = 1.6, 1.6 Hz, H-16b), 5.27 (1H, m, H-10), 7.11 (1H, m, H-11), 7.44 (1H, s, H-5), 9.54 (1H, s, H-7); ^13^C NMR (125 MHz, CDCl_3_) δ 18.0 (CH_3_, C-17), 23.1 (CH_2_, C-13), 30.1 (CH_2_, C-14), 30.5 (CH_3_, C-19), 32.3 (CH_2_, C-2), 45.7 (CH, C-1), 46.5 (CH_2_, C-9), 51.8 (CH_3_, C-21), 76.6 (CH, C-10), 113.6 (CH_2_, C-16), 116.7 (C, C-4), 122.0 (CH, C-5), 134.3 (C, C-12), 144.8 (C, C-15), 147.7 (CH, C-11), 150.6 (C, C-6), 162.9 (C, C-18), 166.6 (C, C-3), 172.9 (C, C-20), 177.1 (CH, C-7), 204.3 (C, C-8); EIMS *m*/*z* 388 [M]^+^, 357 [M−OCH_3_]^+^; ESMS *m*/*z* [M]^+^ 388.1524 (calcd. for C_21_H_24_O_7_, 388.1522), 357.1341 (calcd. for C_20_H_21_O_6_, 357.1338).

### 3.3. Activation of Pancreatic Beta-Cell Proliferation

INS-1 cells (insulinoma rat cells) were seeded at a density of 20,000 cells per well in 96-well plates. Compounds **1**, **2**, rubifolide (**7**) and scabrolide (**8**) were assayed at a final concentration of 0.1 μM in culture medium supplemented with 5.5 mM glucose. Proliferation was measured after 24 h using the BrdU kit (Roche, Mannheim, Germany), following manufacturer’s instructions. At least three independent experiments in triplicate were preformed per compound.

## Figures and Tables

**Table 1:** ^1^H and ^13^C NMR spectroscopic data [500 and 125 MHz, CDCl_3_] of compounds **1** and **2**.

No.	1		2	
	δ_C_, Type	δ_H_ (*J* in Hz)	δ_C_, Type	δ_H_ (*J* in Hz)
1	37.6, CH	3.39, m	39.7, CH	2.46, dddd (2.2, 2.5, 9.5, 11.7)
2	32.9, CH_2_	3.15, m	32.7, CH_2_	a: 2.97, dd (2.5, 14.8) b: 3.19, dd (11.9, 15.1)
3	161.3, C	-	162.1, C	-
4	123.4, C	-	122.7, C	-
5	106.5, CH	6.70, s	106.5, CH	6.80, s
6	156.0, C	-	154.3, C	-
7	74.0, CH	5.29, s	75.6, CH	4.59, s
8	74.3, C	-	73.6, C	-
9	40.5, CH_2_	1.51, m 1.87, dd (5.2, 15.0)	43.0, CH	b: 1.89, dd (11.7, 14.8) a: 2.59, dd (4.2, 14.8)
10	74.3, CH	4.85, dd (5.2, 11.3)	78.4, CH	4.97, m
11	62.9, CH	3.73, s	148.6, C	5.86, s
12	60.6, C	-	136.2, C	-
13	22.5, CH_2_	a: 1.45, m b: 2.44, dd (11.5, 14.7)	21.7, CH_2_	a: 2.14, m b: 2.35, ddd (2.8, 11.9, 15.1)
14	30.4, CH_2_	1.42, m 2.04, m	30.2, CH_2_	1.60, ddd (2.8, 2.8, 15.1) 2.02, m
15	144.2, C	-	146.9, C	-
16	118.0, CH_2_	5.37, s; 5.47, s	117.0, CH_2_	5.19, s; 5.36, s
17	46.6, CH_2_	4.18, br s	47.2, CH_2_	4.12, br s
18	184.7, CH	9.89, s	184.3, CH	9.97, s
19	22.4, CH_3_	1.35, s	19.6, CH_3_	1.41, s
20	172.1, C	-	173.5, C	-

**Table 2:** Selected ^1^H and ^13^C NMR data [CDCl_3_] of leptodiol, lophodiol A, **1**, sinumaximol B and **2**.

	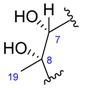			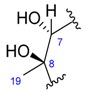	
No.	Leptodiol	Lophodiol A	1	Sinumaximol B	2
δ_H-7_	5.12, br s	5.24, s	5.29, s	4.52, s	4.59, s
δ_H-9_	1.61, dd (8.8, 14.5) 1.68, dd (6.9, 14.5)	1.55, m 1.76, dd (6.4, 14.8)	1.55, m 1.76, dd (6.4, 14.8)	1.85, dd (11.5, 14.5) 2.55, dd (4.0, 14.5)	1.89, dd (11.7, 14.8) 2.59, dd (4.2, 14.8)
δ_H-19_	1.38, s	1.40 s	1.40, s	1.38, s	1.41, s
δ_C-19_	22.7	22.7	22.4	19.8	19.6
δ_C-7_	73.5	73.4	74.0	76.1	75.6
δ_C-9_	41.1	40.9	40.5	43.2	43.0

**Table 3:** ^1^H and ^13^C NMR Spectroscopic data [500 and 125 MHz, CDCl_3_] of compounds **3**, **4** and **5**.

No.	3		4		5	
	δ_C_, Mult.	δ_H_ (*J* in Hz)	δ_C_, Mult.	δ_H_ (*J* in Hz)	δ_C_, Mult.	δ_H_ (*J* in Hz)
1	39.7 CH	2.64, ddd (4.2, 7.1, 7.1)	35.4 CH	2.90, m	45.7, CH	2.70, m
2	49.7, CH_2_	2.44, m 2.46, m	49.9, CH_2_	2.53 m 2.53, m	32.3, CH_2_	3.12, dd (6.6, 14.2) 3.22, dd (8.5, 14.2)
3	215.4, C	-	215.3, C	-	166.6, C	-
4	41.9, CH	2.89, ddd (6.6, 6.6, 10.6)	41.7, CH	2.90, m	116.7, C	-
5	60.8, CH	3.31, d (10.4)	60.9, CH	3.31, d (10.3)	122.0, CH	7.44, s
6	203.5, C	-	205.8, C	-	150.6, C	-
7	55.9, CH_2_	2.68, m	55.9, CH_2_	2.69, m	177.1, CH	9.54, s
8	74.3, C	-	74.3, C	-	204.3, C	-
9	45.6, CH_2_	b: 1.92, m a: 2.34, d (15.1)	45.6, CH_2_	b: 1.90, dd (2.6, 15.4) a: 2.32, d (15.1)	46.5, CH_2_	2.63, dd (7.3, 17.7) 2.99, dd (6.7, 17.7)
10	90.5, C	-	90.3, C	-	76.6, CH	5.27, m
11	155.9, CH	6.76, s	156.0, CH	6.79, s	147.7, CH	7.11, m
12	131.4, C	-	131.1, C	-	134.3, C	-
13	24.0, CH_2_	a: 2.27, ddd (5.4, 9.3, 14.3) b: 2.50, m	22.8, CH_2_	a: 2.28, m b: 2.49, m	23.1, CH_2_	2.16, m 2.25, m
14	31.7, CH_2_	1.89, m	31.5, CH_2_	2.00, m	30.1, CH_2_	1.64, m 1.72, m
15	149.4, CH	-	149.3, C	-	144.8, C	-
16	109.7, CH_2_	4.68, br s; 4.70, br s	114.7, CH_2_	5.00, s; 5.20, dd (0.9, 0.9)	113.6, CH_2_	4.64, br s 4.73, dd (1.6, 1.6)
17	20.9, CH_3_	1.69, s	47.6, CH_2_	4.03, m	18.0, CH_3_	1.68, s
18	17.2, CH_3_	1.04, d (6.6)	17.3, CH_3_	1.04, d (6.6)	162.9,C	-
19	30.1, CH_3_	1.37, s	30.1, CH_3_	1.37, s	30.5, CH_3_	2.21, s
20	170.6, C	-	171.6, C	-	172.9, C	-
O*Me*	-	-	-	-	51.8, CH_3_	3.86, s

**Table 4:** Beta-cell proliferation measurement after treating INS-1 beta-cells with each compound.

Compound (0.1 μM)	Proliferation Ratio ^a^
**1**	2.5 ± 0.65
**2**	2.0 ± 0.61
Rubifolide (**7**)	3.3 ± 0.80 ^b^
Scabrolide D (**8**)	2.8 ± 0.69 ^b^

^a^ Fold change above untreated cells (1.0). A threshold over 1.5-fold was considered to increase proliferation. ^b^ *p* < 0.05 versus control.

## Supplementary Materials

The following are available online at http://www.mdpi.com/1660-3397/16/2/49/s1, Figure S1: ^1^H NMR of **1** in CDCl_3_; Figure S2: ^13^C NMR spectrum of **1** in CDCl_3_; Figure S3: ^1^H NMR of **2** in CDCl_3_; Figure S4: ^13^C NMR spectrum of **2** in CDCl_3_; Figure S5: ^1^H NMR of **3** in CDCl_3_; Figure S6: ^13^C NMR spectrum of **3** in CDCl_3_; Figure S7: ^1^H NMR of **4** in CDCl_3_; Figure S8: ^13^C NMR spectrum of **4** in CDCl_3_; Figure S9: ^1^H NMR of **5** in CDCl_3_; Figure S10: ^13^C NMR spectrum of **5** in CDCl_3_.
