# Supplementary material for: Chloro-Furanocembranolides from Leptogorgia sp. Improve Pancreatic Beta-Cell Proliferation
Source: Mar Drugs. 2018 Feb 2;16(2):49. doi: 10.3390/md16020049 (PMC5852477; doi:10.3390/md16020049)

Supplementary Materials

Chloro-furanocembranolides from *Leptogorgia* sp. improve beta-cell pancreatic proliferation

Amalia B. Gallardo [1,2], Ana R. Díaz-Marrero [1,†], José M. de la Rosa [1], Luis D'Croz [4,5], Germán Perdomo [6], Irene Cózar-Castellano [7], José Darias [1] and Mercedes Cueto [1,*]

[1] Instituto de Productos Naturales y Agrobiología (IPNA-CSIC). Avenida Astrofísico F. Sánchez, 3, 38206 La Laguna, Tenerife, Spain
[2] Facultad de Ciencias, Universidad de Magallanes, Departamento de Química, Avenida Bulnes 01855, Punta Arenas, Chile
[3] Departamento de Biología Marina y Limnología, Universidad de Panamá, Panama
[4] Smithsonian Tropical Research Institute, STRI, Box 0843-03092, Balboa, Panama
[5] School of Health Sciences, University of Burgos, Spain
[6] Instituto de Biología y Genética Molecular (University of Valladolid-CSIC), Spain
* Correspondence: mcueto@ipna.csic.es; Tel.: +34-922-250-144
[†]Present address: University Institute of Bio-Organic Chemistry "Antonio González" (CIBICAN), University of La Laguna, 38206 Tenerife, Spain.

E-mail: mcueto@ipna.csic.es

INDEX:

Figure S1. ^{1}H NMR spectrum of **1** in $CDCl_3$
Figure S2. ^{13}C NMR spectrum of **1** in $CDCl_3$
Figure S3. ^{1}H NMR spectrum of **2** in $CDCl_3$
Figure S4. ^{13}C NMR spectrum of **2** in $CDCl_3$
Figure S5. ^{1}H NMR spectrum of **3** in $CDCl_3$
Figure S6. ^{13}C NMR spectrum of **3** in $CDCl_3$
Figure S7. ^{1}H NMR spectrum of **4** in $CDCl_3$
Figure S8. ^{13}C NMR spectrum of **4** in $CDCl_3$
Figure S9. ^{1}H NMR spectrum of **5** in $CDCl_3$
Figure S10. ^{13}C NMR spectrum of **5** in $CDCl_3$

Figure S1. ^{1}H NMR of **1** in $CDCl_3$

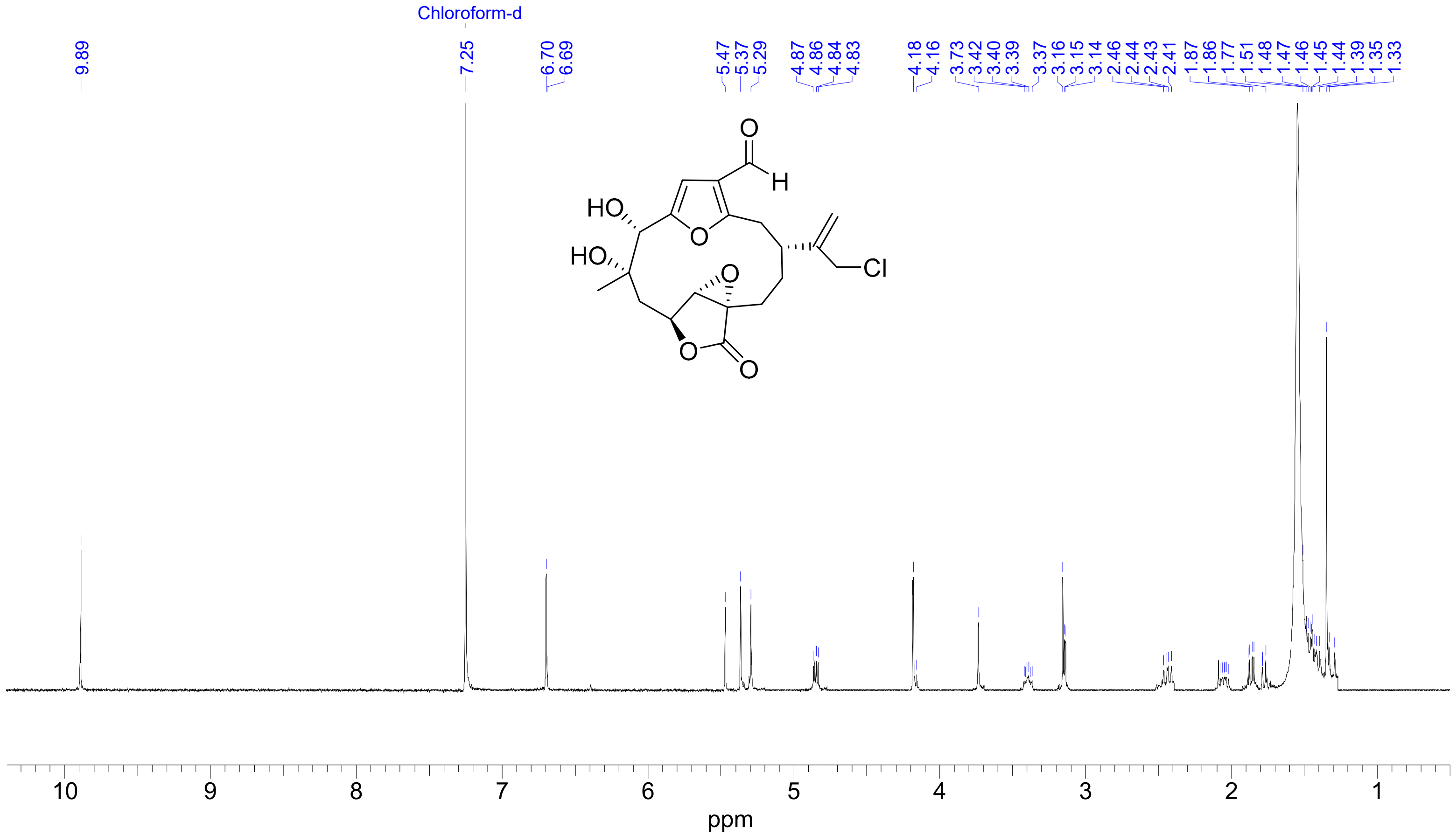

Figure S2. ^{13}C NMR spectrum of **1** in $CDCl_3$

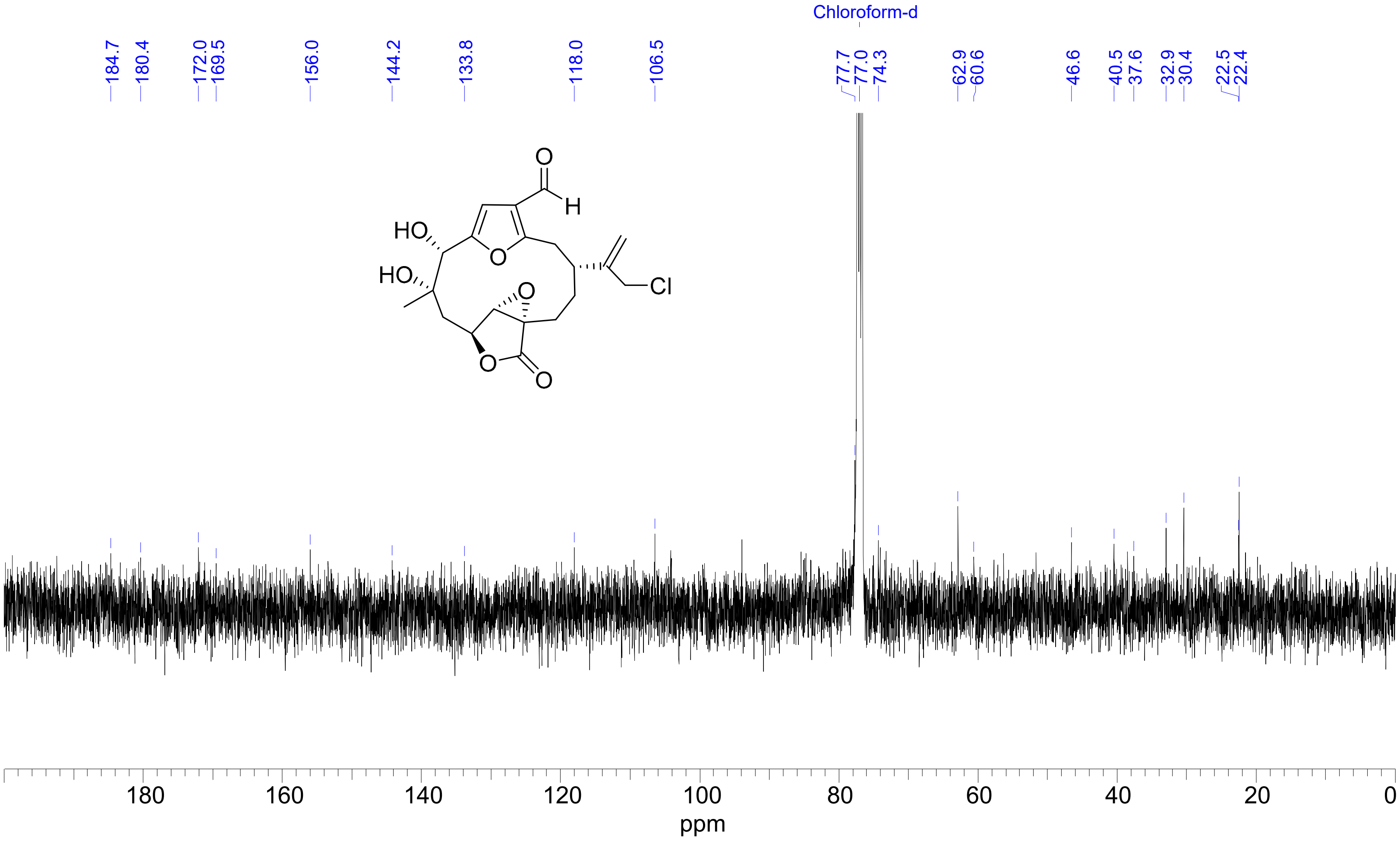

Figure S3. ^{1}H NMR of **2** in $CDCl_3$

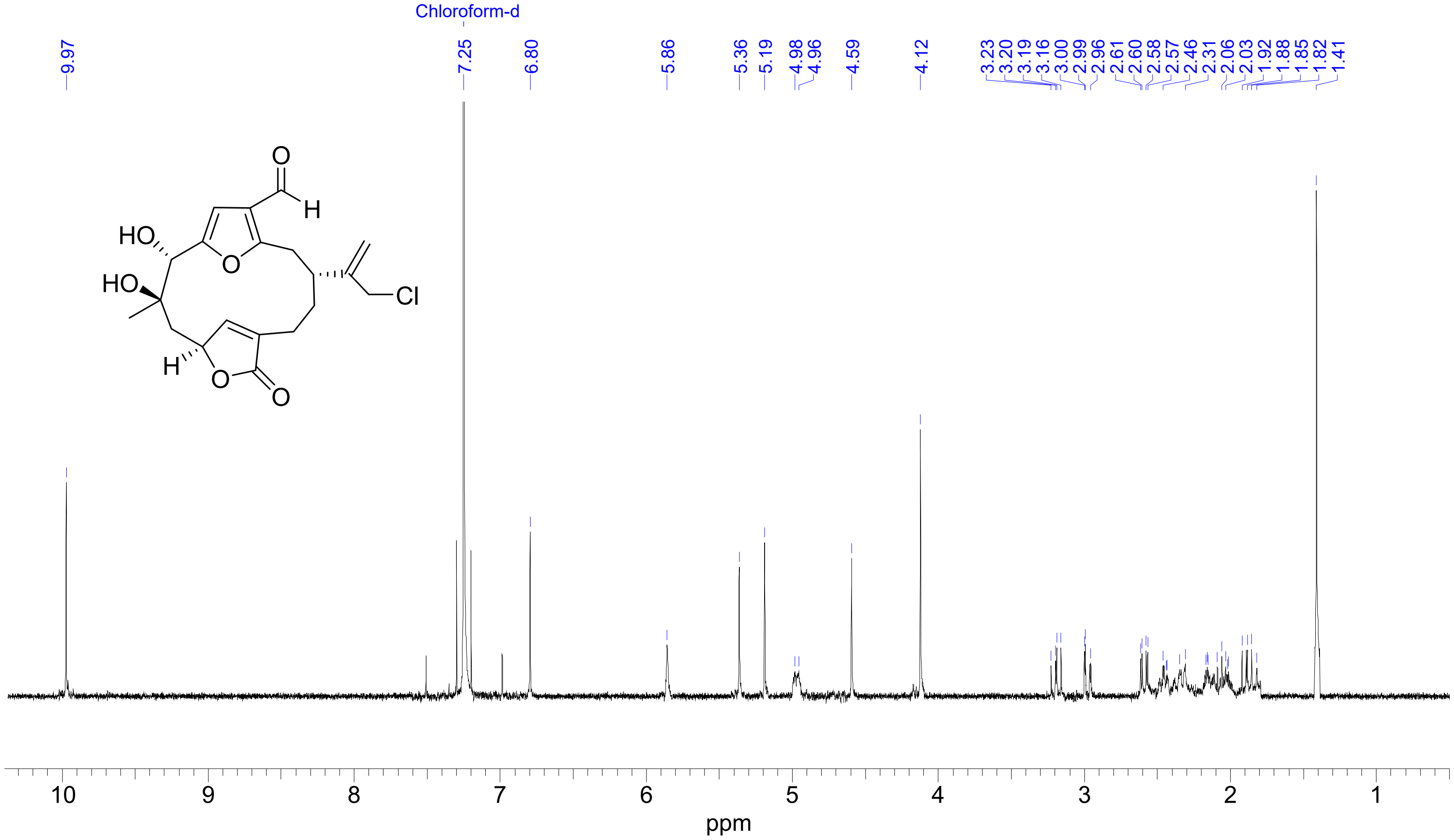

Figure S4. ^{13}C NMR spectrum of **2** in $CDCl_3$

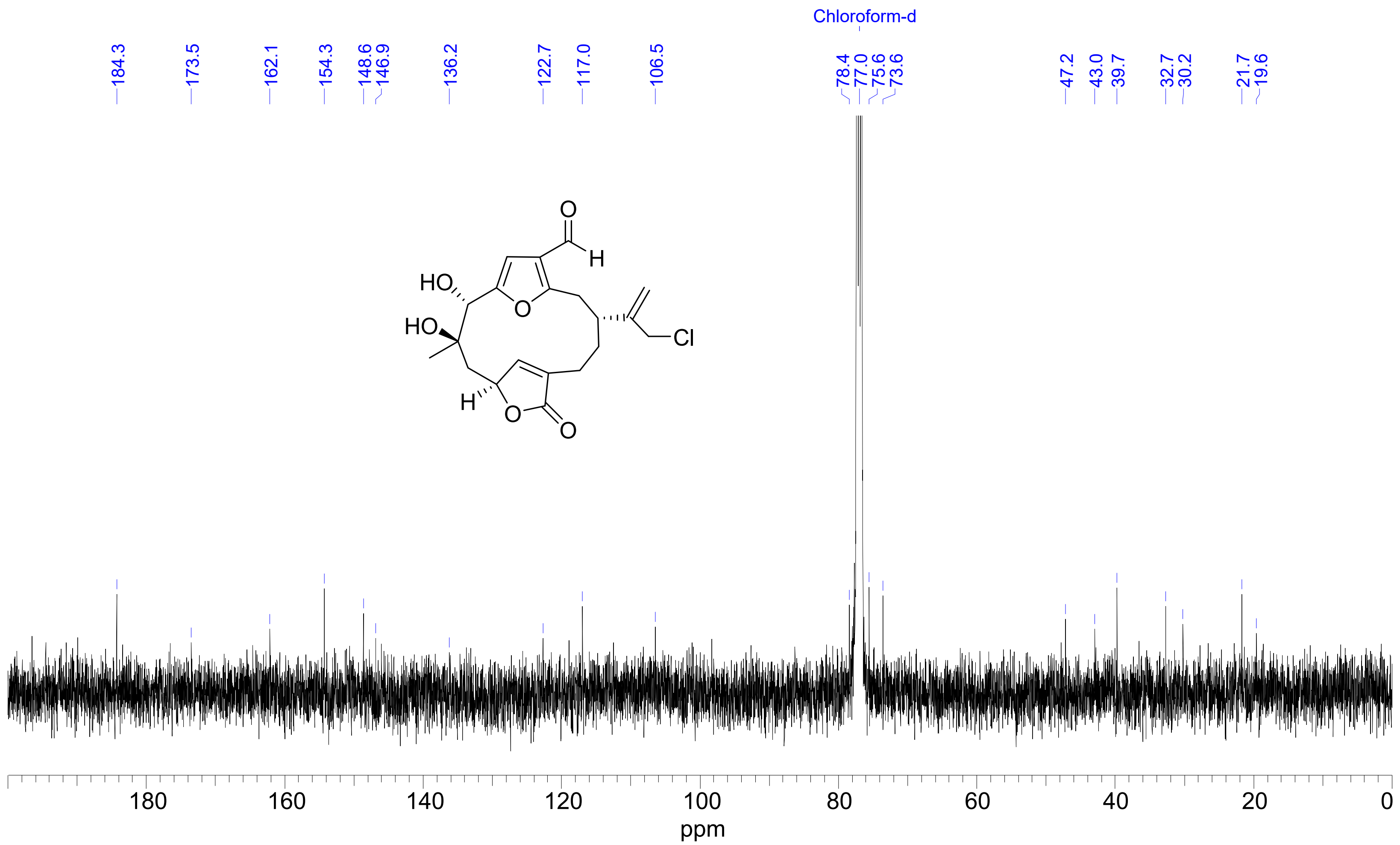

Figure S5. ^{1}H NMR of **3** in $CDCl_3$

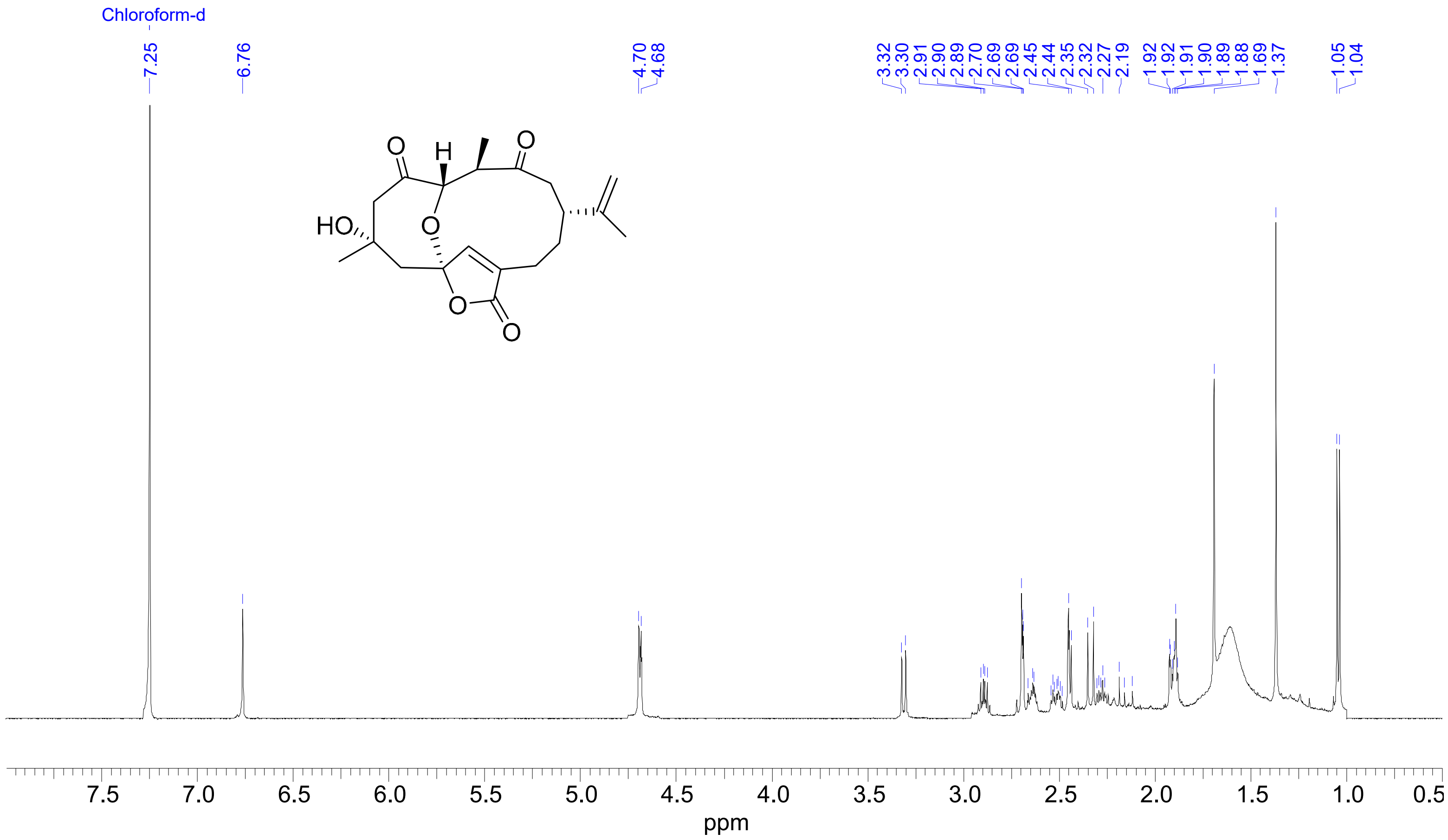

Figure S6. ^{13}C NMR spectrum of **3** in $CDCl_3$

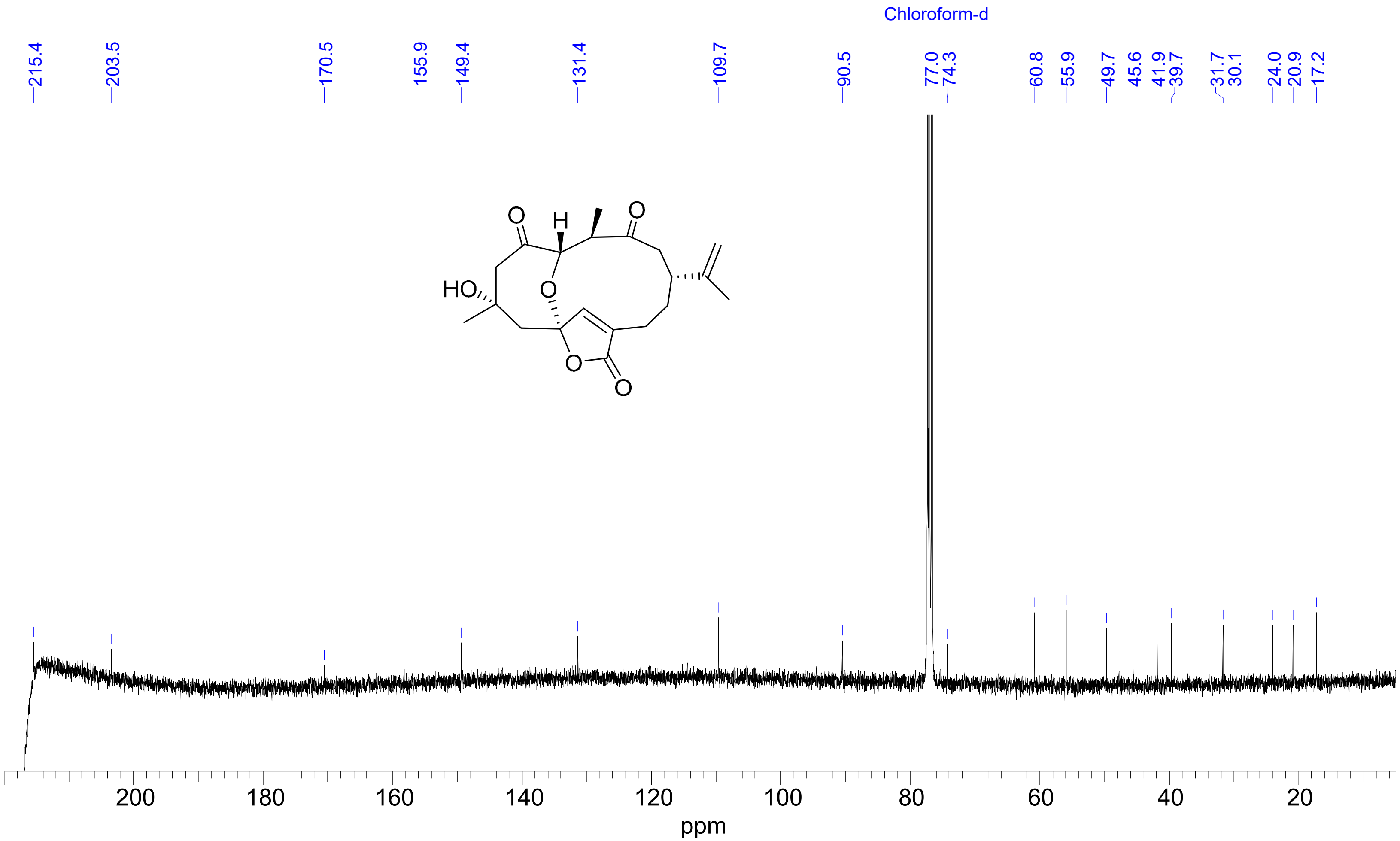

Figure S7. ^1H NMR of **4** in $CDCl_3$

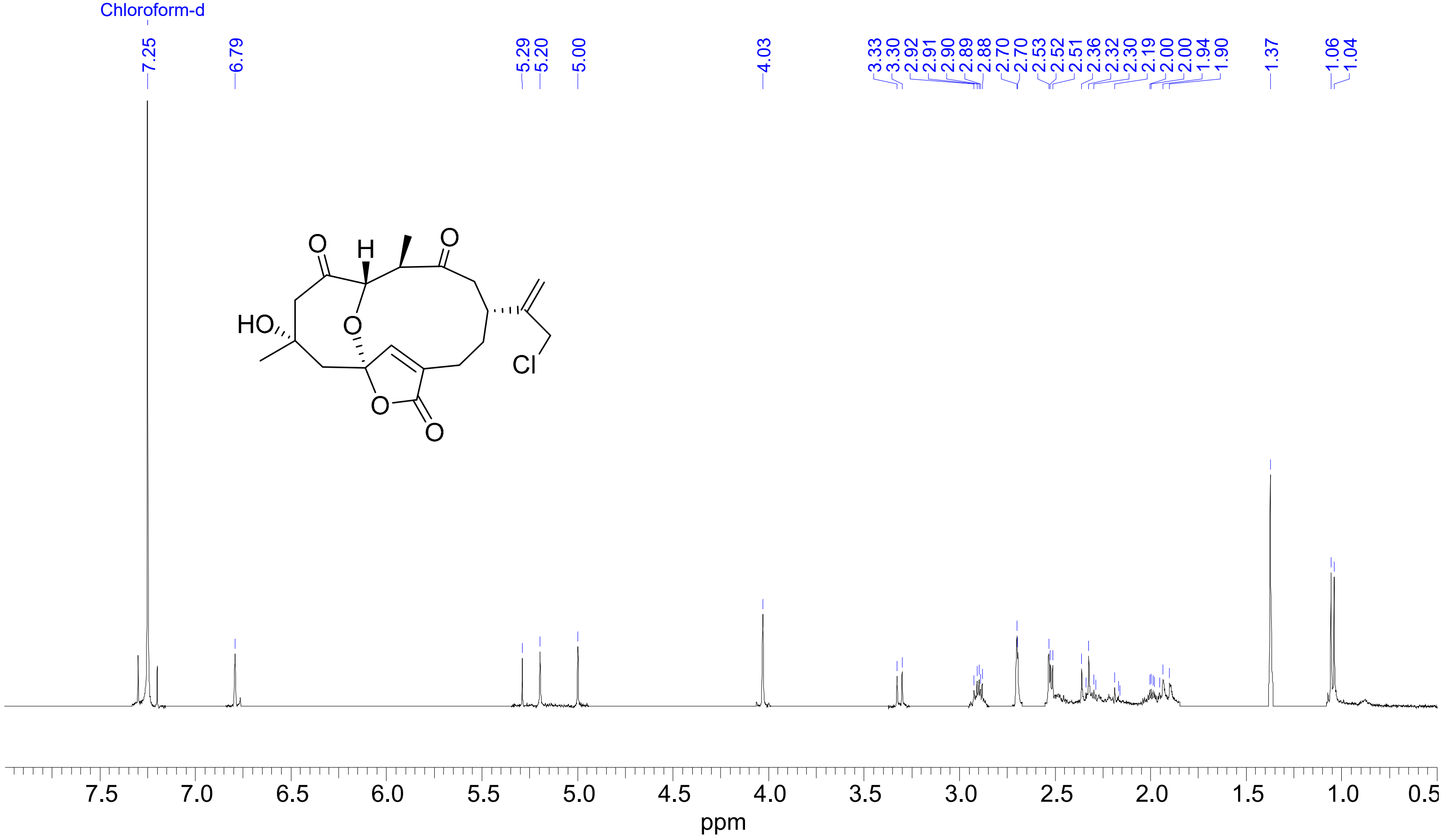

Figure S8. ^{13}C NMR spectrum of **4** in $CDCl_3$

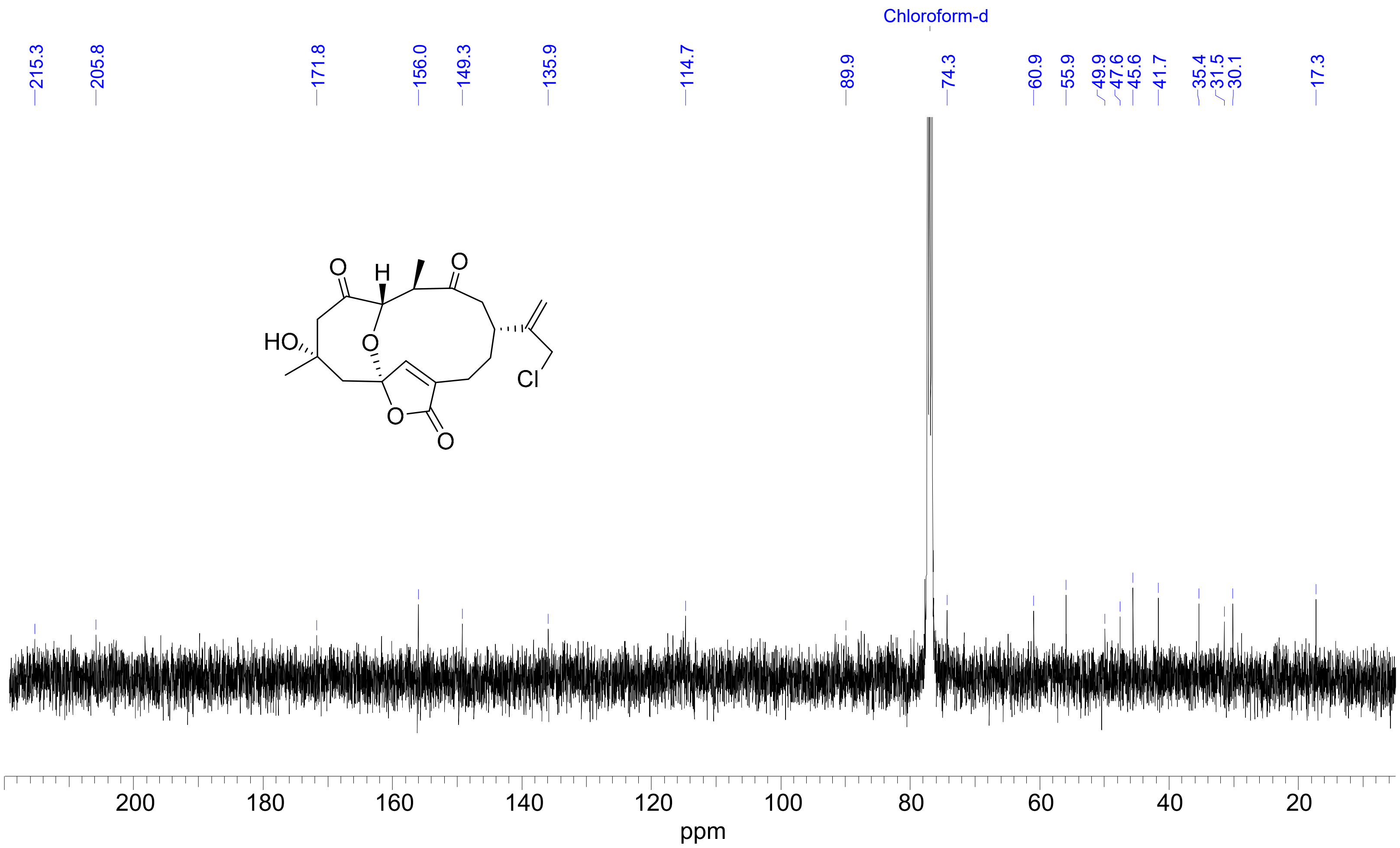

Figure S9. ^{1}H NMR of **5** in $CDCl_3$

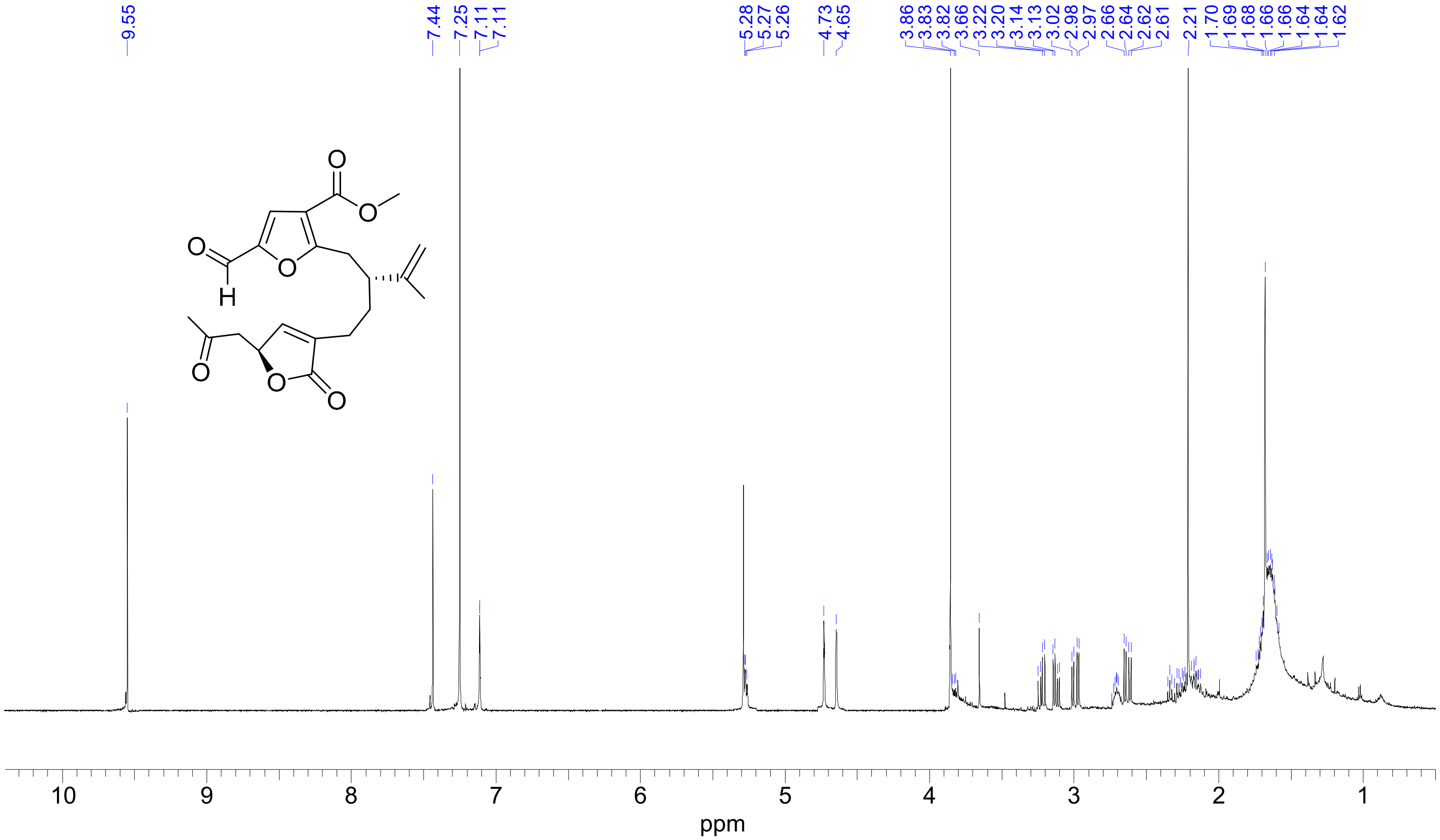

Figure S10. ^{13}C NMR spectrum of **5** in $CDCl_3$

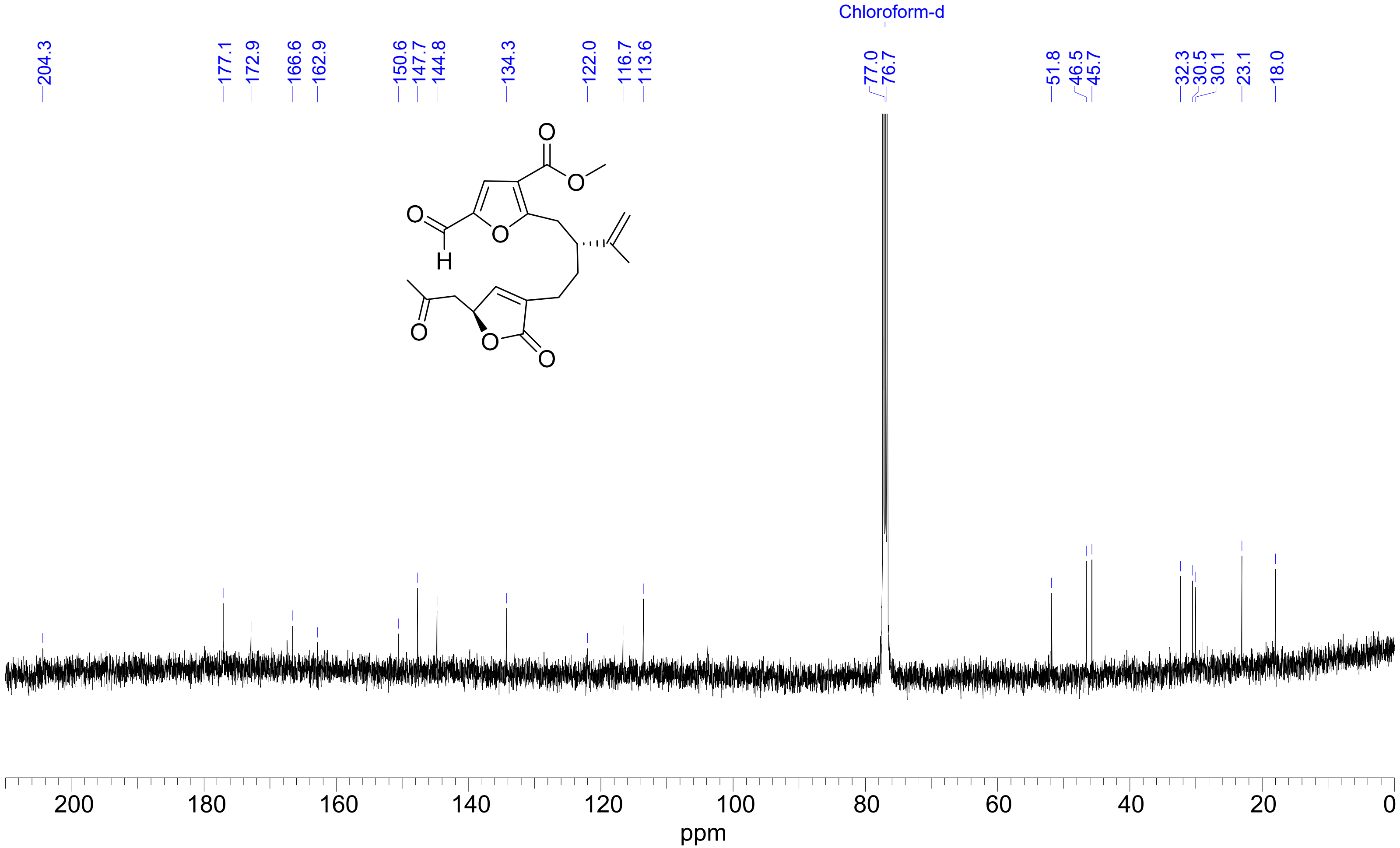

Supplement: Supplementary file 1 [file marinedrugs-16-00049-s001.pdf]
